# Exploring the impact of the reclassification of a hereditary cancer syndrome gene variant: emerging themes from a qualitative study

**DOI:** 10.1007/s12687-023-00644-0

**Published:** 2023-04-03

**Authors:** Laura Wedd, Margaret Gleeson, Bettina Meiser, Rosie O’Shea, Kristine Barlow-Stewart, Amanda B. Spurdle, Paul James, Jane Fleming, Cassandra Nichols, Rachel Austin, Elisa Cops, Melissa Monnik, Judy Do, Rajneesh Kaur

**Affiliations:** 1 School of Clinical Sciences, UNSW Sydney, Sydney, Australia; 2 Centre for Population Genomics, Garvan Institute of Medical Research Sydney, Darlinghurst, Australia; 3 Hunter Family Cancer Service, Newcastle, Australia; 4 Faculty of Medicine and Health, The University of Sydney, Sydney, Australia; 5 Molecular Cancer Epidemiology Laboratory, QIMR Berghofer Medical Research Institute, Brisbane, Australia; 6 Parkville Familial Cancer Centre, Peter MacCallum Cancer Centre and Royal Melbourne Hospital, Melbourne, Australia; 7 Sir Peter MacCallum Department of Oncology, University of Melbourne, Melbourne, Australia; 8 Genetic Services of Western Australia, King Edward Memorial Hospital, Perth, Australia; 9 Genetic Health Queensland, Royal Brisbane and Women’s Hospital, Brisbane, Australia; 10 Adult Genetics Unit, Royal Adelaide Hospital, Adelaide, Australia

**Keywords:** Psychosocial, Variant reclassification, Hereditary cancer syndrome, Lynch syndrome, *BRCA1*, *BRCA2*

## Abstract

The complexity of genetic variant interpretation means that a proportion of individuals who undergo genetic testing for a hereditary cancer syndrome will have their test result reclassified over time. Such a reclassification may involve a clinically significant upgrade or downgrade in pathogenicity, which may have significant implications for medical management. To date, few studies have examined the psychosocial impact of a reclassification in a hereditary cancer syndrome context. To address this gap, semi-structured telephone interviews were performed with eighteen individuals who had a *BRCA1*, *BRCA2* or Lynch syndrome-related (*MLH1*, *MSH2*, *MSH6* or *PMS2)* gene variant reclassified. The interviews were analysed utilising an inductive, qualitative approach and emergent themes were identified by thematic analysis. Variable levels of recall amongst participants were found. Common motivations for initial testing included a significant personal and/or family history of cancer and a desire to “find an answer”. No individual whose uncertain result was upgraded reported negative psychosocial outcomes; most reported adapting to their reclassified result and appraised their genetic testing experience positively. However, individuals whose likely pathogenic/pathogenic results were downgraded reported feelings of anger, shock and sadness post reclassification, highlighting that additional psychosocial support may be required for some. Genetic counselling issues and recommendations for clinical practice are outlined.

**Supplementary Information:**

The online version contains supplementary material available at 10.1007/s12687-023-00644-0.

## Introduction

Genetic testing is routinely offered when there is strong clinical suspicion of a hereditary cancer syndrome, such as hereditary breast and ovarian cancer (HBOC) or Lynch syndrome (LS) (Berliner et al. 2013; Meiser et al. 2020, 2021). Genetic variants are classified using the American College of Medical Genetics and Genomics (ACMG) guidelines, which classify variants into five categories ranging from benign to pathogenic (Richards et al. 2015). Although testing may be uninformative, a likely pathogenic/pathogenic (LP/P) finding can be of benefit since it may allow for a refined risk assessment, appropriate surveillance and risk management, and cascade testing in family members. However, the complexity of genetic variant analysis means that result interpretation is not straightforward, and a proportion of genetic variants will be of uncertain clinical significance (variant of uncertain significance, VUS). Furthermore, changes to our understanding of variant pathogenicity over time mean that a portion of genetic variants will be reclassified (David et al. 2019; Richardson et al. 2022; Savatt et al. 2021).

In the context of hereditary cancer syndromes, recent estimates suggest 3.6–12.4% of variants will be reclassified (Esterling et al. 2020; Mersch et al. 2018; Mighton et al. 2019; Muir and Reagle 2022; Turner et al. 2019). Whilst the majority of reclassifications are not likely to have a significant impact on clinical care, such as a reclassification from LP to P or VUS to likely benign/benign (LB/B), a portion (~ 9–11%) of reclassifications will have a clinical impact (Mersch et al. 2018; Mighton et al. 2019; Muir and Reagle 2022; Turner et al. 2019). An upgrade from VUS to LP/P, for instance, may lead to additional risk-reducing surgeries or surveillance being recommended and allow for the option of cascade testing in family members. By contrast, a downgraded LP/P result will likely lead to an individual being managed based on their personal and/or family history alone, and previously recommended medical management may be revised. For some, irreversible medical decisions may have been made prior to the downgrade (Moghadasi et al. 2016; Turner et al. 2019).

Our understanding of the psychosocial impacts of variant reclassification is only beginning to emerge. Patients with inherited cardiac disease who had their result reclassified were found to have varied emotional reactions to the change in their result; those whose variants were upgraded experienced relief and acceptance, whilst reactions of those whose results were downgraded ranged from relief to disappointment (Wong et al. 2019). Some were also found to misunderstand the implications of the reclassification (Wong et al. 2019). In another study, carriers of a *GALT* variant, which was initially considered pathogenic and causative of the autosomal recessive condition Duarte variant galactosemia, reported few negative psychosocial outcomes after this variant was downgraded to benign (Taber et al. 2018).

Hereditary cancer syndromes present individuals with distinct challenges. Risk management options are available to those identified with a LP/P variant, including irreversible risk-reducing surgeries such as bilateral salpingo-oophorectomy (RRBSO) or prophylactic mastectomy (Meiser et al. 2020, 2021). For those cases where a previously LP/P result has been used to guide clinical management, a downgraded reclassification to VUS or benign has the potential for negative psychosocial outcomes (Turner et al. 2019). Indeed, Halveson et al. in a recent study which gained insight into patient perspectives on variant reclassification, reported that one participant whose *TP53* likely pathogenic result was downgraded post-prophylactic bilateral mastectomy expressed significant anger and resentment at the reclassification (Halverson et al. 2020).

In contrast, individuals who have reportedly received a VUS appraised the possibility of a reclassification positively and hoped for reclassification of their result over time (Solomon et al. 2017). In one case report, the reclassification of a von Hippel-Lindau gene variant from VUS to pathogenic caused some anxiety, but was seen as largely beneficial since it allowed for cascade testing in family members and more effective risk management (Sexton et al. 2015). Tsai et al. reported that individuals who had their cancer susceptibility variant upgraded from VUS to LP/P, largely viewed the reclassification positively, citing that it reduced stress and resolved uncertainty, although a small portion were noted to report negative outcomes (Tsai et al. 2020).

The importance of developing guidelines that support the appropriate clinical management of individuals who experience a clinically significant variant reclassification has been highlighted previously (Loong et al. 2022; Richardson et al. 2022). Whilst such reclassifications are by no means commonplace, the increased uptake of broader gene panel-based testing has led to an increase in reported uncertain findings and therefore reclassifications over time (Esterling et al. 2020). Indeed, a 2020 survey of genetic counsellors from the USA and Canada found that most managed at least one reclassification case per year, with many handling over six cases (Richardson et al. 2022).

Few studies to date have directly explored the psychosocial impact of an upgraded or downgraded hereditary cancer syndrome genetic variant. Studies to date have been limited by small numbers or single case reports and have predominately captured the experience of individuals whose reclassification did not significantly alter their clinical management, for example, a reclassification from LP to P (Halverson et al. 2020; Tsai et al. 2020). Furthermore, to our knowledge, only one has explored this issue in an Australian context (Sexton et al. 2015). The primary aim of this exploratory, qualitative study was to understand the experience of individuals who have had the pathogenicity of a hereditary breast and ovarian cancer (*BRCA1* or *BRCA2)* or Lynch syndrome (LS)-related (*MLH1*, *MSH2*, *MSH6* or *PMS2*) gene variant reclassified, with a particular focus on those who have had a clinically significant change. The findings of this study have been used to inform recommendations for the clinical management of reclassifications.

## Materials and methods

### Participants

Eighteen participants were recruited through five familial cancer centres (FCCs) across Australia. The Peter MacCallum Cancer Centre ethical review board approved the study (HREC/17/PMCC/265). Participants aged 18 and over who had a *BRCA1*, *BRCA2* or LS-related (*MLH1*, *MSH2*, *MSH6* or *PMS2*) germline variant either upgraded or downgraded and were able to provide informed consent were considered eligible for the study. No restriction on the timeframe between participant variant reclassification and study participation was assigned. Clinical genetic counsellors at each recruitment site identified eligible participants and invited them into the study. Potential participants were mailed a letter of invitation, an information sheet and consent form and an opt-in response sheet with a reply-paid envelope. Recruitment responses were returned to the FCC, and those who consented were forwarded to the research team. No survey of those who opted out of the study was conducted.

### Procedures

Participants who opted into the study were contacted by telephone to participate in a semi-structured interview. The interview guide (see Supplemental Material) was developed by LW and reviewed by multiple investigators (RK, BM, RO and MG). Verbal informed consent was obtained prior to the interview. All interviews were conducted by a genetic counsellor, MG, and lasted between 20 min and 1 h and 20 min. Throughout the interview, MG utilised reflective statements to allow for respondent validation. The interviews encompassed issues such as the impact of the reclassification on self, family, cancer-related worry and risk perception. Interviews were professionally transcribed.

### Data analysis

Data was analysed utilising an iterative approach. The first three transcripts were thematically coded by LW and coded independently by RK. Based on this initial coding, a codebook was developed utilising the NVivo software. As additional transcripts were coded throughout the study, any new emerging themes or discrepancies were resolved through discussion to achieve concordance. Emergent themes were identified using the qualitative research framework outlined by Braun and Clarke (2006) (Braun and Clarke 2006). Throughout the analysis, the experiences, emotions, and thoughts of the participants were coded to gain insight into the impact of a reclassification. Once all transcripts were coded and data saturation achieved, LW and MG discussed the study findings to ensure rigor.

## Results

Of the 62 individuals invited to participate, 21 consented (34% response rate). There were no significant differences between participants and nonparticipants in terms of time to variant classification, sex, family history of cancer, personal history of cancer, the reason for an initial testing, gene involved and reclassification (downgrade versus upgrade). A total of 18 individuals completed an interview (29% participation rate), with three who consented lost to follow-up. Interviews were conducted between July 2018 and January 2022. The demographic and medical characteristics of participants are summarized in Table 1. A majority of the sample had a personal history of at least one primary cancer (*N* = 16/18), including breast (*N* = 10), endometrial (*N* = 4), bowel (*N* = 2), ovarian (*N* = 1), gastric (*N* = 1), thyroid (*N* = 1) cancer and osteosarcoma (*N* = 1); one proband had a history of kidney, liver and bladder cancer, in addition to bowel cancer. Initial genetic testing was performed between 1999 and 2018, and the average time to reclassification was approximately 5 years. The majority of participants received an upgrade in the classification of their initial test result (*N* = 13). Of those who received a downgraded result (*N* = 5), four were clinically significant downgrade (pathogenic/likely pathogenic to VUS or benign), whilst one was a downgrade from VUS to likely benign.

**Table 1:** Participant demographics and characteristics (*N* = 18)

	Mean (years)	Range (years)
Age at first cancer diagnosis	48	18–67
Time to variant reclassification	5.1	0.2–18
Age at interview	65	28–90
Time from reclassification to interview	3.7	0.2–13
Variable		
Sex		
Male	4	
Female	14	
Language spoken at home		
English	18	
Other	0	
Highest level of education		
High School	11	
Diploma/certificate	3	
University level	3	
Marital status		
Married or de facto	14	
Not married	4	
Family history of cancer		
First degree relative	14	
Second degree relative only	4	
None	0	
Personal history of cancer		
Yes	16 (6 with multiple primaries)	
No	2	
Reason for initial testing		
Predictive testing	2	
Family segregation studies	1	
Diagnostic testing	15	
Reclassification		
Upgrade (*N* = 13)		
* BRCA1/BRCA2*	6 (VUS to LP/P)	
Lynch syndrome-related	7 (VUS to LP/P)	
Downgrade (*N* = 5)		
BRCA1/BRCA2	3 (LP/P to VUS) 1 (VUS to LB/B)	
Lynch Syndrome-related	1 (LP/P to LB/B)	

*LB/B*, likely benign/benign; *LP/P*, likely pathogenic/pathogenic; *VUS*, variant of unknown significance

Individuals were assigned a unique identifier for data analysis, which reflected whether the reclassified variant was a *BRCA1* or *BRCA2* variant (“BR”) or LS-related (“LS”) and whether the variant was upgraded “U” or downgraded “D”. The unique identifiers are noted following each quotation.

One HBOC participant had multiple variants reclassified, including a *BRCA2* VUS upgraded to LP and two *BRCA1* VUS downgraded to benign. One LS participant had been identified with a pathogenic *MSH2* variant, with no change to classification, along with a *MSH6* VUS which was subsequently upgraded to pathogenic. The interviews for both participants related to their upgraded results, and as such for the purpose of this study have been described in the context of the upgraded variant only.

## Emergent themes

Five key themes were identified: (1) recall; (2) motivations for initial testing; (3) responses and impact of reclassified results, with subthemes of upgraded and downgraded results; (4) genetic counselling and service satisfaction; and (5) communication, comprehension and views of genetic testing.

### Recall

Whilst many participants could broadly recall both their initial genetic test result and their reclassified result, several required prompting throughout the course of the interview and spoke interchangeably of their initial and reclassified result or recounted their experience of their oncology appointments rather than their genetics consults.

A small portion also noted that they found recalling some aspects of their experience challenging: “I don’t remember the fine details, but I remember all the feelings” (BR-U-01) and reflected on the impact that their cancer diagnoses and/or treatment had on their memory:

“I might have looked at it, but I have to be honest, I can’t remember…Yeah, I think I was a little bit out of it. All I was thinking about was trying to get myself better”—BR-D-04, on recalling her initial result

“Um, to be truthful, I can’t really- I can’t remember what you said because yeah, I- um, I was in the middle of operations still”—BR-U-05

A small portion (*N* = 4) had more significant difficulties recounting their experience. One participant could not recall their result as having been upgraded from a VUS to LP but had interpreted her initial VUS as causative. Another had very limited recall altogether of having had genetic testing, “I’d like to know…you said the results have changed, what are the results that have changed?”—LS-D-01.

### Motivations for initial testing

Motivations for initial testing were varied (see Supplementary Table 1 for exemplary quotes). For most, initial genetic testing was guided by a significant personal and family history (*N* = 13/18). For two cases, initial testing was based on a personal history of cancer alone. A large portion (*N* = 9/18) highlighted that their decision to undergo genetic testing was partly, if not wholly, influenced by a medical recommendation. Other motivators for genetic testing reported included a desire to assist in the genetic diagnosis of family members and to take preventative action, along with a desire to “find an answer” for the family.

### Responses to initial and reclassified result

The response to the initial and reclassified result was varied, with participants reporting a range of emotional reactions, including anxiety/worry, minimal impact, disappointment, guilt and disbelief/surprise. This differed substantially between those who received a VUS result that was upgraded over time, and an initial LP/P result that was downgraded. Feelings of relief and gratitude were expressed by those who had their VUS result upgraded, whilst those whose results were downgraded from LP/P expressed anger and sadness.

#### Upgrades: responses to initial uncertain result

Several participants who received an initial VUS described that the result had a minimal impact on them (*N* = 6/13). However, a portion reported feelings of disbelief, disappointment or surprise at the uncertain finding and highlighted that they had expected a causative variant to be found (*N* = 8/13). Others reported feeling anxious or worried. See Supplementary Table 2 for exemplary quotes.

#### Upgrades: responses to reclassification

For those who experienced an upgrade of a previously reported VUS, the majority of responses were positive, with the upgrade resolving uncertainty and providing “an answer”, “Like I know why I’ve got breast cancer and there was no other reason.” (BR-U-01).

Several participants (*N* = 6/13) highlighted the benefit of the upgraded result for their family:

“I’m glad…because of my family more than me…so they don’t through all of this”—BR-U-05

For many, the upgrade of their result was anticipated (*N* = 5/13), and several highlighted that their personal and/or family history of cancer guided their views regarding their future cancer risk, over and above their genetic test result:

“Only that it was interesting to note, that’s all. I knew there must have been something wrong because let’s face it, from the time of my initial testing to the time I received that letter I’d had two more cancers”—LS-U-02

“I say in my own mind, I felt that I already had the gene, it didn’t change any concerns that I had for future risk, or any other cancers or anything so that didn’t change.”—BR-U-01

A common reaction was a relief (*N* = 5/13):

“I actually felt relief that we knew what we’re dealing with.”—BR-U-04

“So, I was relieved to be told, you know one way or the other… so I could get on with my life basically”—LS-U-07

Others (*N* = 3/13) expressed gratitude at the continued work of health services:

“It gave me a bit of comfort actually to know that you’re on top of it.”—LS-U-03

For a minority who received an upgraded result, anxiety and worry (*N* = 4/13) occurred, as the reclassified result led to new information related to other cancer risks being provided:

“I think the thing that surprised me most was my thinking on it afterwards…because I hadn’t thought about my breast cancer for, I don’t know how many years”—BR-U-04

“They sent in the post you know you can get one in so many for brain tumours and all that. That was pretty confronting”—LS-U-04

“I just thought that because I’d had the hysterectomy done and that was it, that I, you know, it wouldn’t pop out with me again”—LS-U-06

Feelings of guilt also occurred (*N* = 2/13):

“I suppose you could say it got to me that I’m passing on all these terrible things to my, to my beautiful girls… it kept on popping in all the time that I was responsible for the death of my beautiful son”—LS-U-06

#### Upgrades: impact to medical decision-making

A majority of participants (*N* = 8) who had their initial uncertain result upgraded to pathogenic did not report any major changes to their screening or preventative surgery as a result of the reclassification, as many already had increased surveillance in place or had preventative surgery based on their personal and family history. One participant, who had undergone bilateral mastectomy prior to any genetic testing, subsequently opted for a RRBSO and hysterectomy post-upgrade.

Several participants reported having undergone risk-reducing bilateral salpingo-oophorectomy (RRBSO), hysterectomy and/or preventative mastectomy prior to receiving any genetic result, highlighting that this occurred at the time of their cancer diagnosis and/or was guided by their personal or family history:

“My breast surgeon when he performed the prophylactic mastectomy and found out they were pre-cancerous cells, he said to me that I should have my ovaries out.”—BR-U-01

Two participants reported electing preventative mastectomy partly based on their VUS but highlighted that “it was the history more than anything” (BR-U-05) which influenced their decision, and “I kind of just distrusted [the result] even though it was unclassified if that makes sense” (BR-U-03).

#### Downgrades: responses to the initial result

For those who received an initial pathogenic or likely pathogenic result, disbelief, shock and disappointment were reported (see Supplemental Table 2)*.* One individual whose pathogenic result was downgraded to benign/likely benign had a very limited recall of their initial test result and was not able to articulate their feelings or reaction, “Sorry but I’ve never been – once they said it stopped with you, I just turned off and nothing’s shown any different since” (LS-D-01).

#### Downgrades: responses to reclassification

##### LP/P to VUS

Four participants had a LP/P result downgraded to VUS or LB/B. For those who could recall their reclassification (*N* = 3/4), all described feeling anger and disbelief at the downgrade:

“How the hell can you test all of our DNA and tell us, okay, this is what’s in your family and then take it away from us?”—BR-D-01

“So, I felt like when I went in and got the original results, we sat down. It was all in writing…whereas afterwards I kind of got off the phone and thought, “I don’t know that I entirely understood.”—BR-D-02

One participant, who was enrolled in a clinical trial, felt anger and worry at the implications the downgrade might have on her eligibility “I was more worried about, I was on this program, and I’d lose it.” (BR-D-04). She also reported feelings of resignation towards the downgrade, “That’s just the way life goes, I suppose…I just have to try and figure out a way to try and get through what I’m doing going through now” (BR-D-04).

However, the participants also reflected on the positive aspects of the downgrade for their family “It’s great news for them. Maybe not great news for me and my sister, but great news for everyone else” (BR-D-02).

Overall, the emotional response for these participants was found to be complex, with unresolved feelings of sadness remaining for one participant: “I try not to spend too much time thinking about it, because it does make me a bit sad… You hate to think that you’ve gone through multiple surgeries for no reason.”—BR-D-02.

##### VUS to LB

One participant (BR-D-03) with a personal history of bilateral breast cancer had her *BRCA2* VUS downgraded to likely benign. The reclassification of the result had a minimal impact overall; “I thought well that’s interesting, at least that’s not a problem that I passed down to my children”. She did not describe feeling disappointed that she did not have a genetic diagnosis; “that didn’t really matter because they can’t know everything at this stage”.

#### Downgrades (LP/P to VUS): medical decision-making and family implications

Whilst one participant had not undergone risk-reducing mastectomy prior to the reclassification, two participants elected to have risk-reducing surgery based on an initial pathogenic result, which was subsequently downgraded:

“That was in part of the decision-making about surgery. So, I knew that I had BRCA1, but it hadn’t been reclassified… my understanding was that there was an increased risk and you know that it was up to me, whether I had a second breast, the second mastectomy prophylactically”—BR-D-02

“We don’t need our ovaries or uterus or anything anymore. That’s the first thing we’re going to get done…I mean we were told we could go through our whole lives and carry the gene and not get cancer but there is quite a high risk getting ovarian or breast cancer with that BRCA gene result.”—BR-D-01 on receiving an initial positive predictive test result

In both cases, the downgrade of their result had a significant impact on their views of their prior medical decision-making, and each described ongoing negative impacts of their surgeries:

“Both my older sister and I have gone through early menopause medically, not naturally. When I think back and I mean six years on, we’re both still going through menopause. We get quite a few symptoms, and it does disrupt your life at times.”—BR-D-01

“I was very pleased for my daughter’s sake [but] it was, was not a good result with the mastectomy and particularly actually with the one that was prophylactic. I’ve had lots of ongoing problems.”—BR-D-02

This impact extended to relatives who had also received positive predictive results that were subsequently re-classified, as well as family members for whom predictive testing was no longer an option:

“[My niece] had almost looked into the fact of going through IVF and storing her eggs and discarding the ones that were positive to the gene…. [She said] ‘My God, I would have discarded those eggs for nothing’”—BR-D-01

“I felt bad telling my sister…I sort of felt like, I wish I hadn’t been so quick to tell you about the original result, because maybe a few months might have made a difference, and then you might not have had to go through all the surgery.”—BR-D-02

### Genetic counselling and service satisfaction

#### Pre-test counselling and result disclosure

Pre-test counselling and results disclosure varied for participants. The majority who received an initial uncertain result reported being informed of the possibility of a variant reclassification, whilst the majority who received an initial pathogenic finding were not aware this was a possibility prior to receiving their reclassified result. However, many participants had difficulty recalling the information provided at the time of initial testing.

Individuals were frequently contacted about their reclassified result by clinical services via phone or letter in the first instance, with the offer of a follow-up consultation (VUS to LP/P *N* = 6/13, LP/P to VUS/LB/B *N* = 2/4), and a portion reported receiving only a phone call or letter (VUS to LP/P *N* = 4/13, LP/P to VUS/LB/B *N* = 1/4, VUS to B *N* = 1/1). Overall, participants were satisfied with the mode of result delivery (*N* = 12/18). However, some who were contacted via an unscheduled phone call indicated that either a scheduled phone call or an in-person appointment was their preferred mode of delivery, including two participants whose results were downgraded:

“I think a phone call’s good because it has that personal touch…. The only thing I’d say…I mean, not expecting to get phone calls like that…I had friends visiting. I was throwing clothes on as I was talking on the phone”—BR-U-06

For some, however, face-to-face results disclosure was preferred, along with a follow-up letter “so you had it as information” (BR-U-05). This was particularly the case for those who received a downgraded result**,** “I think probably the one on one, not like to be told over the phone”—BR-D-01.

#### Service satisfaction

A majority of participants were satisfied with the information and support provided by genetic services (*N* = 14/18), describing that they “couldn’t have been more wonderful” (LS-U-03), and that “the geneticists that I went through are absolutely brilliant, beautiful and answer any questions” (LS-U-04). Six participants highlighted that they felt they had the ongoing support of services and could recontact them if needed, “I feel if I, if I wanted to, I could ring her up today…and talk to her about it.” (BR-U-04).

However, four participants described certain aspects of their care that did not meet their needs: “Initially [when we] were told the new findings, I guess we felt a bit left in the lurch.” (BR-D-01), “I think they need something…softened about it you know, not just to go and say well this is what it is” (LS-U-04).

Some reflected that they did not feel they had open communication with the genetic service post-results disclosure, including one participant whose result was downgraded from pathogenic:

“I sort of felt like it was like a closed file, that’s right, this is great news. We don’t need to worry about you anymore” (BR-D-04)

### Communication, comprehension and views of genetic testing

#### Family communication

A majority of participants communicated both their initial (*N* = 15/18) and reclassified (*N* = 17/18) results to family members. One participant, who did not communicate her initial uncertain result, reported telling the family once the result was upgraded “I had something concrete that I needed to tell them rather than it could- there’s a possibility.” This choice was made, in part, to minimise distress in the family “I didn’t want to alarm anybody or talk to anybody about it until I found out definitely what was going on” (LS-U-05). Other reasons for not disclosing results included a lack of contact with family members, “Because I haven’t got a clue where they are” (BR-U-04), as well as a perception that they would be disinterested in the upgraded result, “I didn’t because they paid so little attention to me the first time- I told my son and my daughter but I didn’t tell my brother and his family” (BR-D-03).

Overall, participants did not report significant difficulty with communicating results in the family. Some reported difficulty communicating uncertain findings or when they did not feel that they had adequate information provided by clinical services, “Well, this is what I’ve heard, but I can’t tell you exactly, because I don’t have the information.” (BR-D-02). Whilst others described that communicating the results raised feelings of guilt, and was emotionally challenging, “I tried to make it as I suppose light as possible and that we did not cry and carry-on about anything…but it came as a shock to them too” (LS-U-06).

#### Understanding and views of genetic testing

Overall, the majority of participants viewed genetic testing positively (*N* = 14/18). Genetic testing was described as a “fantastic tool” (BR-D-02), which “saves people’s lives” (BR-U-02), and empowered decision-making. Whilst some described that knowledge of their genetic result caused worry, the majority highlighted that, overall, it was “better to know” (LS-U-06), as this allowed them and at-risk family members to proactively engage in screening and/or risk-reducing surgery (*N* = 7):

“It’s far better to know you have the potential to develop a problem…while you’re young enough to adjust your lifestyle to lessen the risk”—LS-U-07

This attitude was similarly reflected by individuals in the cohort who received a downgraded result:

“A genetic test is a vital clue on how to treat it or diagnose it, it’s definitely a positive to help not only us, but research as well.”—BR-D-01

No interviewee reported negative feelings towards genetic testing. Some, however, held a more neutral or indifferent view towards testing, describing that whilst it might be useful for family members, their circumstance meant it had little impact on them directly.

“There’s nothing wrong with trying to find out things… maybe it’s better for those that do need it”—BR-D-04

Five participants reflected on having difficulty understanding genetic concepts; “it was rather probably confusing knowing about all this genetic thing, you know, you sort of often don’t understand it” (LS-U-01), “it was a little bit above my head of what was going on” (LS-U-06). For one participant their understanding of genetics and recall of testing was limited to the extent that they could not articulate their attitudes towards genetic testing. All individuals in this category had attained a high school (years 10–12 equivalent) level of education.

## Discussion

Few studies have directly explored the psychosocial impact of a genetic variant reclassification, particularly in the hereditary cancer context. Our findings provide insight into the experiences of those who have had a clinically significant reclassification (*N* = 17/18, 94%) that led to changes in medical management and risk advice for the individual and/or his or her family.

### Recall

Previous studies described that not all individuals who have their results reclassified accurately recalled or interpreted the new finding. Wong et al. found that a portion of individuals misunderstood the clinical implication of their reclassified result and in one example, inappropriately ceased medical treatment post downgrade. Halverson et al. reported that overall, there was a low level of comprehension and recall of interviewees who had their hereditary cancer syndrome result reclassified. In this study, many participants could generally recall their reclassified result; however, they often had difficulty recalling specific aspects of their experience. Whilst the recall issues for our interviewees do not appear to be as significant as those reported by Halverson et al. we found that a portion of interviewees had significant recall issues, to the extent they could not remember their results and misunderstood their reclassification.

Overall, there did not appear to be a strong relationship between the length of time between the initial result to reclassification and/or interview and poor recall. The average time to reclassification in our study was 5.1 years, with an average of 3.7 years from reclassification to interview. Similar average lengths of time were reported by Halverson et al.; however, we note greater variations in the length of time within our group of participants when compared to the study reported by Wong et al.; for instance, one participant in our study had their variant reclassified 18 years after their initial result, and for 33%, 5 years or more had passed between the reclassification and interview.

Several other factors are known to impact patient comprehension and recall of genetic test results including health literacy and reduced information retention at the time of a cancer diagnosis (Halverson et al. 2020; Lillie et al. 2007; Wing et al. 2021). Those who had a poor recall in this study typically had a high school level of education or less and were over 70 years. This suggests that multiple factors likely influenced recall issues in this group; however, the small numbers of individuals included and the qualitative methodology used mean that this fining cannot be generalised.

### Responses to a reclassified result

Individuals whose reclassified genetic result does not directly lead to changes in their clinical care or is in the context of a disease that has a milder clinical course have been reported to generally have neutral or minimal reactions to a reclassification (Halverson et al. 2020; Taber et al. 2018). In our study, those who experienced an upgrade of a VUS, which was largely anticipated and desired, viewed their reclassification positively. They typically adapted to their reclassified result easily, which resolved uncertainty, validated past medical decision-making, and improved care for their relatives. Similar findings have been reported elsewhere, suggesting that those with upgraded results experience fewer adverse psychosocial outcomes (Tsai et al. 2020; Wong et al. 2019).

Negative psychosocial outcomes and feelings such as anger and disappointment have been previously reported in cases where a genetic variant has been downgraded from LP/P to VUS or benign (Tsai et al. 2020; Wong et al. 2019). Three interviewees in this study reported that both they themselves and their family felt anger at the loss of their genetic diagnosis and experienced worry and unresolved feelings of sadness and guilt. These findings suggest that when a reclassification is unexpected and not in line with the individual’s understanding of their past medical or family history, or where irreversible medical decisions have been made in the interim, patients may not adapt to downgraded results as easily. Individuals have been reported to be more vulnerable to psychological distress when results are unexpected; instances where individuals have limited or inaccurate recall of their initial result, or where appropriate pre-test counselling regarding the possibility of a reclassification has not occurred, may, therefore, be at increased risk for adverse psychosocial outcomes (Meiser et al. 2016). Further research into the psychosocial impact of downgraded genetic results, in the context of both predictive testing and diagnostic testing, is required to understand these impacts further.

### Service satisfaction

General satisfaction with the information and support provided by genetic services was reported by the majority of participants. However, a small number reported that they did not feel they could openly communicate with genetic services and would have benefited from psychosocial support or more clarity around risk information. The challenges faced by clinical services regarding the follow-up of patients with new information or a revised result have been discussed previously; genetic counsellors have highlighted the significant resourcing issues that make a longer-term follow-up of patients difficult (Muir and Reagle 2022; Vora et al. 2022). However, possible recall issues, difficulty adjusting to new results or communicating results to family members mean that some who experience a clinically significant variant reclassification warrant further support by services. Services must be adequately resourced to provide this support.

### Views of genetic testing post reclassification

Altogether, no interviewees reported negative views of genetic testing after their reclassification, including those who experienced a downgrade of a LP/P result. Genetic testing was seen as a beneficial tool, which can empower decision-making and provide families with an answer, views which have been reported elsewhere (Halverson et al. 2020; Tsai et al. 2020). No individual was found to mistrust their genetic result altogether post reclassification. Being informed of the possibility of a reclassification prior to testing was desired, reiterating recommendations that clinicians should discuss the possibility of a reclassification with patients as part of pre-test counselling (Wong et al. 2019). Appropriate pre-test counselling and discussion around the evolving nature of genetic variant interpretation with patients prior to reclassification may assist in minimising adverse psychosocial outcomes and feelings of shock at the time of results disclosure (Mighton et al. 2021).

## Recommendations

The experiences of interviewees in this study highlight the importance of establishing recommendations for the management of genetic variant reclassifications in a hereditary cancer context. Overall, appropriate pre-test counselling, which emphasises the possibility of a reclassification at the time of initial testing, should be provided routinely. Recall issues and misunderstanding of the implications of the reclassified result have been reported elsewhere and have been observed in a portion of individuals in this study, highlighting the need for clinicians to confirm that a patient’s conceptualisation of their result is accurate (Wong et al. 2019; Halverson et al. 2020). Given that negative psychosocial outcomes may occur for those who experience a downgrade, face-to-face consultation and, at a minimum, a single follow-up consultation should be offered. Genetic services must be appropriately resourced to provide this support.

## Study limitations

Overall, the recruitment of participants into this study, particularly for those who received a downgraded result, was challenging. The participation rate (29%) in this study was low; however, no significant differences were found in terms of sociodemographic, medical and family history variables. All individuals recruited had a significant personal and/or family history of HBOC or LS, and as such our findings may not be reflective of affected probands without a family history or those who have had a predictive genetic test result downgraded. We did not survey individuals who opted out of the study, and as such cannot ascertain whether the lack of recruitment related to negative experiences of a reclassification. Also, it is possible that clinical services hesitated to invite participants into the study where adverse reactions occurred. Our findings highlight that downgraded reclassifications of pathogenic variants can be related to negative psychosocial responses. However, we did not formally assess the psychosocial needs or responses of individuals in this study, and as such further research is required. The small sample size also limits the ability to compare the experience of individuals with HBOC and LS. Furthermore, significant recall issues, which occurred for some participants, may have impacted on their ability to accurately recount aspects of their experience, such as pre-test counselling and medical decision-making. Finally, given the qualitative nature of the methodology, no generalisations can be made.

## Conclusion

Our study reports on the experience of individuals who have had a hereditary cancer syndrome genetic variant upgraded (VUS to LP/P) or downgraded (LP/P to VUS to LB/B). Most individuals were found to have few negative psychosocial outcomes, adapted to their reclassified result readily and appraised their genetic testing experience positively. However, a portion reported negative psychosocial outcomes, particularly where irreversible medical decisions had been made in the context of a downgraded pathogenic variant, and the reclassified result was found to provoke worry, anxiety, anger and disbelief. In these cases, additional psychosocial support was desired, highlighting that genetic services must be appropriately resourced to provide ongoing support to individuals who have a genetic variant reclassified. Further research, particularly in cases of downgraded genetic results, is needed to enable the development of clear guidelines for the clinical management of reclassification cases.

## Supplementary Information

Below is the link to the electronic supplementary material.

Supplementary file1 (DOC 81.0 KB)

## Data Availability

The interview guide is submitted as supplemental material.

## Declarations

### Animal studies

This article does not contain any studies with human or animal subjects performed by any of the authors.
