# Supplementary material for: Exploring the impact of the reclassification of a hereditary cancer syndrome gene variant: emerging themes from a qualitative study
Source: J Community Genet. 2023 Apr 3;14(3):307–17. doi: 10.1007/s12687-023-00644-0 (PMC10272031; doi:10.1007/s12687-023-00644-0)
Supplement: Supplementary file 1 — Supplementary file1 (DOC 81.0 KB) [file 12687_2023_644_MOESM1_ESM.doc]

**Supplemental material**

**Exploring the impact of the reclassification of a hereditary cancer syndrome gene variant: emerging themes from a qualitative study**

Laura Wedd,^1,2^ Margaret Gleeson,^3^ Bettina Meiser,^1^ Rosie O’Shea,^4^ Kristine Barlow-Stewart,^4^ Amanda B. Spurdle,^5^ Paul James,^6,7^ Jane Fleming,^4^ Cassandra Nichols,^8^ Rachel Austin,^9^ Elisa Cops,^6^ Melissa Monnik,^10^ Judy Do,^1,2^ Rajneesh Kaur^,1,4^

^1^School of Clinical Sciences, UNSW Sydney, Australia; ^2^Centre for Population Genomics Garvan Institute of Medical Research Sydney Darlinghurst Australia; ^3^Hunter Family Cancer Service, Newcastle, Australia; ^4^Faculty of Medicine and Health, The University of Sydney, Sydney, Australia; ^5^Molecular Cancer Epidemiology Laboratory, QIMR Berghofer Medical Research Institute, Brisbane, Australia; ^6^ Parkville Familial Cancer Centre, Peter MacCallum Cancer Centre and Royal Melbourne Hospital, Melbourne, Australia; ^7^Sir Peter MacCallum Department of Oncology, University of Melbourne, Melbourne, Australia ^8^ Genetic Services of Western Australia, King Edward Memorial Hospital, Perth, Australia; ^9^Genetic Health Queensland, Royal Brisbane and Women’s Hospital, Brisbane, Australia; ^10^Adult Genetics Unit, Royal Adelaide Hospital, Adelaide, Australia;

Corresponding author: Prof Bettina Meiser; email: b.meiser@unsw.edu.au

**Semi-structured interview guide**

**First genetic result**

1. Can you tell me about your experience of receiving your **first** genetic result? NOTE: Not your reclassified result

Probe: Why did you have genetic testing? What were your expectations of genetic testing?

1. How did you feel about your future cancer risk when you received this result?
2. What was the advice given to you about medical management at the time?
3. What did you decide to do, based on your result? Probe: did you have any surgery? Additional screening? Did your genetic test result influence this decision?
4. Did you tell your family about this genetic result?

Probe: Did anyone else in your family have genetic testing following your initial result?

1. Did anyone explain to you the possibility that your genetic test result might change or be reclassified? If so, was this before you had genetic testing?

Probes:

Yes – can you tell me what you were told?

No – what information would you liked to have been told?

What do you think of the information you were given before your reclassification? Too much or too little?

**Reclassification of result**

1. Can you tell me about your experience of your reclassification?

Probe: How did your genetic result change? What was the reason for the reclassification?

1. How were you told about of your reclassification?

Probe: Was it a telephone call/email/letter? Do you think this was the best way? Would you have preferred a different approach?

1. What was your initial reaction to your reclassification?

Probe: Was the reclassification a good thing, or a bad thing? How did it make you feel about your future cancer risk?

1. Have you told your family about your reclassified genetic result? If so, how did you find this? Probe: was it difficult or easy to tell them about the new result? Why?

**Decision-making**

*Tailored interview guide:*

*Upward reclassification (e.g. uncertain to pathogenic)*

10a) After you received your reclassified result, what was the advice given to you about medical management? What did you decide to do?

11a) Did any family members decide to have genetic testing? How do you feel about this?

*Downward reclassification (e.g. pathogenic to uncertain)*

10b) After you received your reclassified result, how did you feel about your treatment decisions / the treatment decisions you had already made?

11b) What impact did this reclassification have on family members who decided to have genetic testing? (if any)

**Genetic testing and reclassification**

12) Considering your overall experience of your reclassification, what are your feelings towards genetic testing?

13) Based on your experience what went well, or did not go well during the reclassification process? Probe: Do you consider your reclassification to be a positive or negative experience? Why?

14) How do you view health professionals after your reclassification experience? Probe: What support would you like them to provide?

Table 1: Motivations for initial testing

| Theme | Exemplar quote(s) |
| --- | --- |
| Influenced by medical recommendation | *“My doctor thought, because there was a family history of two close relatives, that I should have it done”* *–* BR-D-03  *“In one breath, they decided to do some genetic screening, because it was, you know, because of my previous history.*”*–* BR-D-02 |
| Facilitate a genetic diagnosis for family members | *“Well, once my eldest granddaughter she had, when they found out about her, they sent her to a geneticist...I just made it clear that anything I could do to help in any way that he was just to let me know” –* LS-U-06 |
| Preventative action | *“If we can prevent anything from happening to us, we’re right in there” –* BR-D-01 |
| Desire to find an answer | *“I guess we're really trying to find out what was happening, why all these girls were being diagnosed with breast cancer in my family” –* BR-U-06 |

Table 2: Responses to initial result

| Direction of reclassification | Theme | Exemplar quote(s) |
| --- | --- | --- |
| Upgrades | Minimal impact | *“I wasn't on high alert or anything…I just remember it not being that important and so I put it on the backburner sort of thing. I thought no more of it”* (BR-U-04). |
|  | Disbelief, surprise | *“Knowing my family history had, you know, my mother and my brother especially had passed away with cancer, I was of course, quite concerned…and probably when it was [said] it was inconclusive, I was surprised.”* (LS-U-01).  “*Thinking because of [my] family history…I thought, ‘Yes, this is going to be the case.’ … and they said, ‘No, it's not.*’” (LS-U-06). |
|  | Disappointment | *“I was disappointed that it wasn't more definitive at that stage.”* (BR-U-06) |
|  | Uncertainty | *“If there's unknown significance you know you can't really do anything”* (BR-U-06), *“[we’re] left at a dead end at the moment”* (BR-U-02). |
|  | Anxiety, worry | *“I knew something wasn’t right… but they hadn’t got to the crux of the matter…it was still to me and them a mutated gene.”* (LS-U-04).  *“You know it started ringing alarm bells for us, because you know my – we’ve got predominantly girls in my family”* (BR-U-02). |
| Downgrades | Disbelief | *“I was kind of blindsided because it wasn't something I was expecting”* (BR-D-02). |
|  | Disappointment | *“I wasn't very happy at that afterwards, and I just put all that information away.”* (BR-D-04) |
